# Explanation from Dr. Harlan

**Published:** 1839-07-20

**Authors:** R. Harlan


### FOREIGN CORRESPONDENCE.

### EXPLANATION FROM DR. HARLAN.

To the Editors of the Medical Examiner.

Library of the Institute,

*Paris, May 27th*, 1839.

I hasten to correct an error which occurs in my last letter, describing M. Magendie's discovery on the nervous functions, which ought to have been thus rendered into English:

"The sensitive and motor nerves of the spine, when both remain untouched, are equally sensible.

"When the sensitive nerves are cut, the motor nerves immediately lose their sensibility.

"If we cut through the middle of the motor nerves, the end which remains attached to the spinal marrow is altogether insensible; the opposite end preserves, on the contrary, an extreme sensibility. In this case sensibility departs from the circumference to the centre.

"If we cut the sensitive nerves at their middle part, the end attached to the spinal marrow is very sensible; the end attached to the ganglion, on the contrary, has lost all sensibility."

M. Magendie proposes to determine if this influence of the sensitive nerves upon the motor nerves be maintained in the spinal marrow between the various fasciculi which compose it, and which themselves may be distinguished into *sensitive* and motor.

R. Harlan.

